# A generalized deep learning network for fractional anisotropy reconstruction: Application to epilepsy and multiple sclerosis

**DOI:** 10.3389/fninf.2022.891234

**Published:** 2022-08-05

**Authors:** Marta Gaviraghi, Antonio Ricciardi, Fulvia Palesi, Wallace Brownlee, Paolo Vitali, Ferran Prados, Baris Kanber, Claudia A. M. Gandini Wheeler-Kingshott

**Affiliations:** ^1^Department of Electrical, Computer and Biomedical Engineering, University of Pavia, Pavia, Italy; ^2^NMR Research Unit, Department of Neuroinflammation, Queen Square Multiple Sclerosis Centre, UCL Queen Square Institute of Neurology, University College London (UCL), London, United Kingdom; ^3^Department of Brain and Behavioural Sciences, University of Pavia, Pavia, Italy; ^4^Department of Radiology, IRCCS Policlinico San Donato, Milan, Italy; ^5^Department of Biomedical Sciences for Health, Universitá degli Studi di Milano, Milan, Italy; ^6^Department of Medical Physics and Bioengineering, Centre for Medical Image Computing (CMIC), University College London, London, United Kingdom; ^7^E-Health Center, Universitat Oberta de Catalunya, Barcelona, Spain; ^8^Brain Connectivity Centre, IRCCS Mondino Foundation, Pavia, Italy

**Keywords:** deep learning, fractional anisotropy, diffusion weighted MRI, reduced acquisition time, temporal lobe epilepsy, multiple sclerosis

## Abstract

Fractional anisotropy (FA) is a quantitative map sensitive to microstructural properties of tissues *in vivo* and it is extensively used to study the healthy and pathological brain. This map is classically calculated by model fitting (standard method) and requires many diffusion weighted (DW) images for data quality and unbiased readings, hence needing the acquisition time of several minutes. Here, we adapted the U-net architecture to be generalized and to obtain good quality FA from DW volumes acquired in 1 minute. Our network requires 10 input DW volumes (hence fast acquisition), is robust to the direction of application of the diffusion gradients (hence generalized), and preserves/improves map quality (hence good quality maps). We trained the network on the human connectome project (HCP) data using the standard model-fitting method on the entire set of DW directions to extract FA (ground truth). We addressed the generalization problem, i.e., we trained the network to be applicable, without retraining, to clinical datasets acquired on different scanners with different DW imaging protocols. The network was applied to two different clinical datasets to assess FA quality and sensitivity to pathology in temporal lobe epilepsy and multiple sclerosis, respectively. For HCP data, when compared to the ground truth FA, the FA obtained from 10 DW volumes using the network was significantly better (*p* <10^−4^) than the FA obtained using the standard pipeline. For the clinical datasets, the network FA retained the same microstructural characteristics as the FA calculated with all DW volumes using the standard method. At the subject level, the comparison between white matter (WM) ground truth FA values and network FA showed the same distribution; at the group level, statistical differences of WM values detected in the clinical datasets with the ground truth FA were reproduced when using values from the network FA, i.e., the network retained sensitivity to pathology. In conclusion, the proposed network provides a clinically available method to obtain FA from a generic set of 10 DW volumes acquirable in 1 minute, augmenting data quality compared to direct model fitting, reducing the possibility of bias from sub-sampled data, and retaining FA pathological sensitivity, which is very attractive for clinical applications.

## Introduction

Diffusion weighted (DW) imaging is a magnetic resonance (MR) method sensitive to the movement of water molecules within the tissue, thus providing information about the integrity of brain microstructure *in vivo*. This technique is largely employed for investigating microstructural changes in the brain caused by, e.g., neurodegenerative or neurological diseases (Jones, 2011).

From DW images, several computational models have been proposed to investigate the microstructure of the brain. The simplest model is the diffusion tensor (DT) (Basser et al., 1994; Lope-piedrafita, 2018), which has been used to extract metrics to quantify microstructural changes in the tissues, particularly white matter (WM) (Fortin et al., 2017). The DT models the diffusion process as a symmetrical second-order tensor for each voxel. Theoretically, to measure the full DT, six non-collinear diffusion-encoding directions are needed, i.e., six DW volumes, each obtained with a different DW gradient direction, plus one non-DW volume (b_0_). In practice, it has been demonstrated that such a limited number of DW directions may introduce biases in the maps and that increasing the number of DW volumes (e.g., to 35 or 60) (Landman et al., 2007; Zhan et al., 2011) results in less noisy and biased DT estimates. This inevitably leads to longer acquisition times of the order of several minutes, which limits the clinical adoption of DT imaging. Several quantitative maps can be obtained from the DT, with fractional anisotropy (FA) (Basser, 1995) being widely used due to its sensitivity to changes in tissue microstructure (Alexander et al., 2007; Giannelli et al., 2010).

Previous studies showed that deep learning (DL) can be a valid method to reduce the number of DW volumes required to generate quantitative diffusion maps (Golkov et al., 2016; Li et al., 2019). In these studies, the input to the model was either a single voxel or cubes of 3 x 3 x 3 voxels. Our model, instead, used as input all the brain voxels within a slice to capture the global context. It is worth noting that an alternative method that can provide FA maps with a short acquisition time uses anatomical T1-weighted (T1-w) images as input of a generative adversarial network (Gu et al., 2019). This method, however, generates a completely synthetic FA map that has not been shown to be sensitive to pathology.

A similar recent study (Aliotta et al., 2021) used DL to map FA from three DW volumes acquired with a consistent acquisition scheme, fixed b-values and diffusion-encoding directions, on a single scanner, and was not tested on existing datasets, different from the training one, in the presence of pathologies.

In our study, we aimed to jump a step forward by implementing a U-net DL network that can: (1) map from a small number of DW volumes (i.e., 10, equating to an acquisition time of 1 min) to FA, a microstructural map well known in clinical context; (2) take advantage of datasets with high-geometrical and DW angular resolution with corresponding high-quality FA maps for training the network, e.g., using the Human Connectome Project (HCP) data (Van Essen et al., 2013); (3) tackle the generalization problem given a network trained with a specific dataset and offer applicability to clinical datasets from different scanners without retraining; and (4) retain sensitivity to pathology. We validated the network with unseen data from the HCP dataset and with two datasets from temporal lobe epilepsy (TLE) and multiple sclerosis (MS) studies, acquired with different protocols on different scanners.

## Methods

### Subjects

#### HCP dataset

Pre-processed images of 100 healthy controls (HC) scanned for the HCP were downloaded from the Connectome DB (http://db.humanconnectome.org) (Van Essen et al., 2013). After a visual quality check, 24 of these subjects were discarded because of severe artifacts, such as phase-encoding EPI distortions in the cerebellum, not corrected by the standard HCP pre-processing pipeline. The remaining 76 subjects (43 women, 29.41 ± 3.62 years) were used to develop the network.

#### TLE dataset

A first retrospective dataset was used to test the performance of the network. Eighty-four subjects were selected within those recruited for an Italian multi-center research project on TLE. Subjects were divided, clinically into three groups: 34 HCs (16 women, 31.97 ± 7.73 years), 21 TLE patients with the epileptogenic zone in the left hemisphere (LTLE; 13 females, 33.13 ± 11.28 years), and 29 TLE patients with the epileptogenic zone in the right hemisphere (RTLE; 17 females, 37.97 ± 9.86 years) (Gaviraghi et al., 2021).

#### MS dataset

A second retrospective dataset, collected at the University College London, was also used to test the performance of the network. The dataset included images of 29 HCs (19 women, 34.58 ± 10.23 years), 18 patients with clinically isolated syndrome (CIS; 12 women, 49.01 ± 7.16 years), 63 patients with relapsing–remitting multiple sclerosis (RRMS; 48 women, 47 ± 7.58 years), and 13 patients with secondary progressive multiple sclerosis (SPMS; 9 women, 47.83 ± 7.79 years) (Brownlee et al., 2019).

### MR acquisition and pre-processing

#### HCP dataset

MR images were acquired using a customized Siemens 3T Connectome Skyra scanner with a dedicated gradient insert (W. U. Minn Consortium Human Connectome Project, 2017). The DW acquisition included a spin-echo EPI sequence with TR = 5520 ms and TE = 89.5 ms. We downloaded DW data with minimal pre-processing (EPI distortion, eddy current, and subject motion correction plus realignment to standard Montreal Neurological Institute (MNI) space) at a resolution of 1.25 x 1.25 x 1.25 mm^3^ and a matrix size of 145 x 174 x 145. The DW acquisition included 288 volumes: 18 volumes acquired with b-value b = 0 s/mm^2^ (b_0_) and 270 volumes acquired with b = 1000/2000/3000 s/mm^2^ (90 non-collinear DW directions for each b-value). The acquisition time of the diffusion protocol was calculated to be over 27 min based on the repetition time and the number of volumes acquired.

The T1-w data were acquired with a 0.7 x 0.7 x 0.7 mm^3^ resolution and co-registered to the DW data (to obtain a resolution of 1.25 x 1.25 x 1.25 mm^3^).

#### TLE dataset

MR images were acquired using a Siemens 3T MAGNETOM Skyra scanner with standard gradients. The DW imaging protocol included a spin-echo EPI sequence with TR = 8400 ms and TE = 93 ms, 96 DW volumes with b = 1000/2000 s/mm^2^ (48 non-collinear DW directions per b-value), and 13 b_0_ volumes. The spatial resolution was 2.24 x 2.24 x 2.2 mm^3^, and the matrix size was 100 x 100 x 96. The acquisition time of the diffusion protocol was around 16 min.

The scanning protocol also included a high-resolution 3D T1-w volume (resolution 1 x 1 x 1 mm^3^).

#### MS dataset

MR images were acquired using a 3T Philips Achieva MRI scanner. The DW imaging protocol included a spin-echo EPI sequence with TR = 14000 ms, TE = 82 ms, 60 volumes with b = 300/711/2000 s/mm^2^ (8/15/30 DW per b-value), and 7 b_0_ volumes. The spatial resolution was 2.286 x 2.286 x 2.5 mm^3^, and the matrix size was 96 x 96 x 60. The acquisition time of the diffusion protocol was around 16 min.

The scanning protocol also included a volumetric T1-w imaging sequence (resolution 1 × 1 × 1 mm^3^).

For the clinical datasets, TLE and MS, the pre-processing steps included denoising, Gibbs ringing artifact, EPI distortion, eddy current, and subject motion correction.

For all datasets, we computed the ground truth (GT) FA, using all DW volumes (from here referred to as STANDARD method) fitted with the diffusion kurtosis model (Ades-Aron et al., 2018) because this model has a better accuracy than the DT model (Veraart et al., 2011). We also calculated FA, considering only 10 volumes, using the DT model (from here referred to as the Reduced STANDARD method) (Behrens et al., 2003). All fitting procedures were based on weighted linear least-squares algorithms.

### Network design

To train our network, we used the HCP dataset. The 76 subjects were divided as follows: 54 for the training set, 11 for the validation set, and 11 for the test set. Each slice of each subject was considered separately from the rest of the data, but all slices of a subject were used only in the set they belong to (training, validation, or test).

The network input consisted of a set of 10 DW images of each slice, where each DW image was used as one of 10 input channels, with a single output channel corresponding to the FA map of the same slice. Details of how the input–output pairs were constructed are given here below.

#### Input

We reduced the number of DW images from 288 to 10: one b_0_ and nine with b = 1000 s/mm^2^ (Jensen and Helpern, 2010).

We selected the 10 DW by randomly sampling one volume of the 18 b_0_ and nine volumes out of the 90 DW directions with b = 1000 s/mm^2^, using the Camino toolkit (Cook et al., 2005). We used the command “subsetpoints” to divide the 90 points in DW space, i.e., the b-vector coordinates, into subsets that are equally spread over the sphere using simulated-annealing optimization to search for the best configuration. We split the 90 DW volumes with b = 1000 s/mm^2^ into 10 different subsets, hence providing 10 different possible input datasets for each slice of each subject.

We aimed to create a network that is independent of the DW directions used for training so that it can be applied to any dataset with nine DW directions and one b_0_, independent of the exact b-vector coordinates. We, therefore, trained the network with different combinations of DW subsets as explained later in the training section.

We cropped the image background around the brain to have a slice matrix size of 128 x 160 voxels. We normalized the intensity of the data of each subject separately, considering each input set of 10 DW volumes together. We then set the voxels outside the brain to zero.

#### Output

As ground truth (GT), we used the FA map calculated from 288 DW volumes, i.e., STANDARD method. The same cropping and background nulling performed to the input images were applied.

We adapted the 2D U-net architecture (Ronneberger et al., 2015) implemented in TensorFlow (Keras). The convolutional layers in the network were set to 20. The number of filters was, respectively, 64, 64, 128, 128, 256, 256, 512, 512, 256, 256, 128, 128, 64, and 64.

### Network training

#### Tuning of hyperparameters

We selected the best set of hyperparameters as the combination that minimized the mean root mean square error (RMSE) on the validation set.

These included batch normalization (Ioffe and Szegedy, 2015), dropout (Srivastava et al., 2014), activation function, loss function, λ of the L2 regularization method, and batch size (Keskar et al., 2017):

- Batch normalization—We evaluated the network without any batch normalization layer and then added a batch normalization layer after each convolution layer.
- Dropout—We evaluated the network without any dropout layer and we added a dropout layer with a probability of 0.5 after the last two convolution layers of the encoder (4 e 5).
- Activation function—We evaluated sigmoid against rectified linear unit (ReLU) (Glorot et al., 2011; Maas et al., 2013) activation functions in the output layer; the ReLU activation function was used on all other layers.
- Loss function—We considered three different functions:

1. The Mean Square Error (MSE)
  $$\begin{array}{l} L_{MSE}\;=\;\frac{1}{\;n}\overset{n}{\underset{i=0}{\sum }}{\left({Ŷ}_{i}\;-\;Y_{i}\right)}^{2} \end{array}$$
2. The Root Mean Square Error (RMSE)
  $$\begin{array}{l} L_{RMSE}\;=\;\sqrt{\frac{1}{\;n}\overset{n}{\underset{i=0}{\sum }}{\left({Ŷ}_{i}\;-\;Y_{i}\right)}^{2}} \end{array}$$
3. The sum of RMSE and the Structural Dissimilarity Index (DSSIM) (Hou et al., 2021).
  $$\begin{array}{l} L\;=\;L_{RMSE}\;+\;L_{DSSIM} \end{array}$$
The DSSIM, derived from the structural similarity index measure (SSIM) (Wang et al., 2004), compares local patterns of pixel intensities that have been normalized for luminance and contrast. We tested whether adding DSSIM enhanced the performance of the network by taking into account the structural detailed information of neighboring voxels. The range of DSSIM is [0, 1] and larger values indicate greater differences.
  $$\begin{array}{l} L_{DSSIM}\;=\;\frac{\left(1\;-\;SSIM\;\right)}{2} \end{array}$$
in which SSIM can vary between −1 and 1 and is defined as follows:
  $$\begin{array}{l} SSIM\left(y,\;ŷ\right)\;=\;\frac{\left(2\mu_{y\;}\mu_{ŷ}\;+\;c_{1}\right)\;\left(2\;\sigma_{yŷ}\;+\;c_{2}\right)}{\left(\mu_{y}^{2}\;+\;\mu_{ŷ}^{2}\;+\;c_{1}\right)\;\left(\sigma_{y}^{2}\;+\;\sigma_{ŷ}^{2}\;+\;c_{2}\right)} \end{array}$$
where *y* and ŷ are two input patches to compare; μ_*y*_ and μ_ŷ_ are the mean pixel intensities of the patches that estimate luminance; $\sigma_{y}^{2}$ and $\sigma_{ŷ}^{2}$ are the respective variances that measure contrast; and σ_*yŷ*_ is the covariance of y and ŷ indicating the structure. The variables *c*_1_ = ${\left(k_{1}L\right)}^{2}$ and *c*_2_ = ${\left(k_{2}L\right)}^{2}$ ensure numerical stability, and L is the dynamic range of the pixel values, while k_1_ and k_2_ are constants. We used L = 1, *k*_1_ = 0.01, and *k*_2_= 0.03.

- λ of the L2 regularization method—We added a regularization term to the loss function, choosing the L2 regularization method to avoid overfitting. We considered three different values for the coefficient λ = 0.0001, 0.001, and 0.01.
- Batch size—We evaluated performance with batch sizes of 16, 24, 50, 64, and 128.

We used the Adam optimizer (Kingma and Ba, 2017) with a small initial learning rate of η=0.001 for setting the network parameters (weight and bias). We used an early stopping rule for terminating training if the loss function did not improve after 10 consecutive epochs.

#### Training on different number of DW subsets

The choice of DW input images is crucial for estimating FA (Hasan et al., 2001). We therefore trained the network, after setting the hyperparameters, four times by changing the network inputs, that is, by increasing the number of DW subsets per slice per subject from 1 to 2, 4, or 7 subsets, each subset being entered as an independent input and associated with the same FA output. This resulted in four trained networks to evaluate against the GT FA maps. When assessing the performance of each network on all test subjects (all unseen by the training), we selected as input, in turns, each of the 10 DW subsets, therefore testing results either on subsets included or not included in the network training, assessing generalizability.

### Quantitative evaluations on network performance and network selection

We selected the RMSE, mean absolute error (MAE), and SSIM (Wang et al., 2004) calculated between the GT FA and the network FA output within the brain as performance metrics.

For each test subject, we provided each one of the 10 subsets, one at a time, as input to the network and thus obtained the estimated network FA; this step was performed for each of the four trained networks.

We assessed whether one of the networks demonstrated less dependency from the choice of the input DW volumes, that is, whose performance metrics had similar values when tested using either DW subsets included in the training or not.

In order to evaluate the advantages of the network FA in terms of fidelity to GT FA and image quality in general, we also calculated the FA from one subset of 10 DW images using the standard model-fitting method.

We calculated the performance metrics for the brain and for WM only, between:

- the GT FA and the FA calculated with the network;
- the GT FA and the standard FA calculated from the reduced (10) DW images.

We performed a statistical test (Mann–Whitney U-test) comparing for each subject the RMSE obtained between GT and network FA with the RMSE obtained between GT and FA calculated in the standard method with only 10 volumes.

### Clinical adoption potential

#### Data curation of clinical studies datasets

Our ultimate aim was to assess whether our best network, as evaluated on the HCP data, can be used clinically, on data acquired with a limited number of DW volumes and with a limited resolution compared to the HCP data; moreover, we wanted to test whether our network maintains or improves the quality of FA compared to datasets with more DW directions and whether it retains sensitivity to pathological changes.

We used two different existing datasets of neurological conditions, i.e., TLE and MS, to assess the network performance.

These two independent datasets have a spatial resolution that is much lower than that of the HCP dataset. DW images were resampled to match the HCP resolution using FSL FLIRT with sinc interpolation (FMRIB's Linear Image Registration Tool) (Jenkinson and Smith, 2001; Jenkinson et al., 2002). After resampling, the images were then pre-processed in the same way as the HCP dataset.

The total number of DW directions was different for each dataset, according to the diffusion protocol used. Using Camino (command “subsetpoints”), we selected a single subset of nine most non-collinear DW directions for each dataset that minimized the electrostatic energy of the points in the DW subset (Jansons and Alexander, 2003). We then randomly selected a b_0_ volume to add to the nine DW volumes and created our input for the network. For the TLE dataset, the nine DW volumes were chosen from those with a b-value equal to 1000 s/mm^2^, while for the MS dataset, the b-value was equal to 711 s/mm^2^.

With these subsets of 10 DW volumes from each clinical study, we obtained network FA maps for each subject. We also calculated FA from the full DW dataset and for the subset of 10 volumes using the standard model-fitting methods.

To qualitatively evaluate the FA obtained with the different methods, we showed images of each FA. In addition, we calculated histograms of the STANDARD method FA values and the FA obtained with 10 DW in the two methods. This was used to assess whether the FA calculated with the 10 volumes had systematic biases compared to the FA used as reference (STANDARD method).

#### Network FA performance on clinical tasks

To assess whether the network FA maintains sensitivity to pathology, we compared FA values in WM, given that FA changes in neurological conditions are mostly reported in WM (e.g., Yap et al., 2013).

For the HCP data (test subjects only), the T1-w images were already co-registered with the respective DW images; consequently, we obtained the WM mask from the T1-w volume using MRtrix3 (Tournier et al., 2019) and applied it to the FA map.

In subjects with TLE and MS, we first segmented the WM mask from the T1-w volume, registered the T1-w volume to DW space, and then applied the transformation to the WM mask to be able to overlap it to FA. This chain of operations was performed with MRtrix3 (Tournier et al., 2019) and FSL FLIRT (FMRIB's Linear Image Registration Tool) (Jenkinson and Smith, 2001; Jenkinson et al., 2002). In patients with MS, FA was calculated in the normal-appearing WM (NAWM) by excluding lesions from the WM mask where it is well known that diffusion anisotropy is altered (Cercignani and Gandini Wheeler-Kingshott, 2019). Lesions were considered as a separate mask also used for extracting metrics. We separated the contributions as the comparisons were made on the averaged values of each subject; in this pathology, the amount of lesions and the size of them vary greatly from subject to subject.

We then compared WM FA obtained with the network with WM FA obtained in the standard method from all DW directions. Analyses were conducted at two levels: individual subject level and group level.

At the individual subject level, we compared histograms of the distribution of WM FA values between the two methods.

For the group-level analyses, we calculated the mean value of WM (or NAWM) FA for each subject and performed between-group statistics to test whether the network FA maintained sensitivity to pathology. We then performed the Mann–Whitney U-test for pairs of clinical sub-groups between the mean values of WM FA either calculated in the standard way or with the network. For the TLE dataset, the comparisons were between HC and LTLE, HC and RTLE, and LTLE and RTLE. For the MS dataset, the comparisons were between HC and CIS, HC and RRMS, HC and SPMS, CIS and RRMS, CIS and SPMS, and RRMS and SPMS. For the MS dataset, we considered NAWM for the patients and also calculated the mean FA value in the lesion mask of subjects with one or more lesions. For each dataset, we performed the Bland–Altman analysis to describe the agreement between the mean WM FA values of the two methods.

## Results

### Network design and training

**Figure 1:**
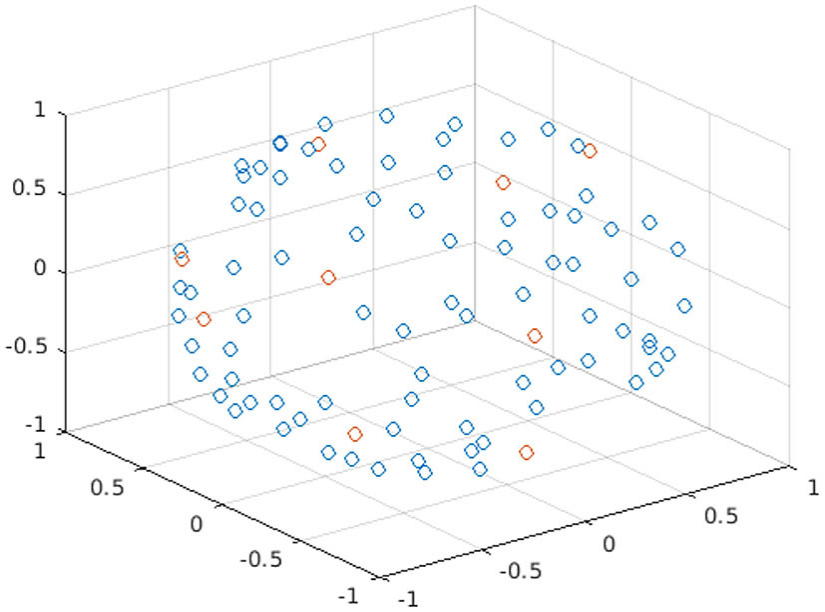
Plot of the spatial coordinates of the b-vectors for the 90 points of b = 1000 s/mm^2^ of the HBP dataset. Each point in the plot represents a different b-vector; the points in red are the b-vectors of one subset selected with Camino.

Figure 1 shows the spatial coordinates for the different diffusion gradient directions, i.e., the b-vectors, for the 90 b = 1000 s/mm^2^ of the HCP data; an example of nine points of a DW subset selected with Camino is highlighted in red.

We successfully obtained FA maps from training the proposed U-net. Training took between 24 and 120 h, depending on the number of training subsets, on an NVIDIA Tesla T4 GPU.

The network provided the best results with this set of hyperparameters: including batch normalization, dropout, with ReLU as the activation function for the last layer, using the sum of *L*_*RMSE*_ and *L*_*DSSIM*_ as loss function, λ = 0.001 for the L2 regularization, and a batch size of 64. The number of epochs was 76.

**Figure 2:**
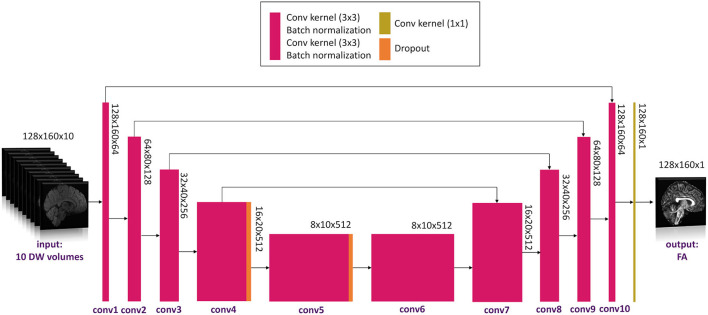
Network architecture used. Conv, convolution.

The network architecture is shown in Figure 2.

### Quantitative evaluation on network performance and network selection

**Figure 3:**
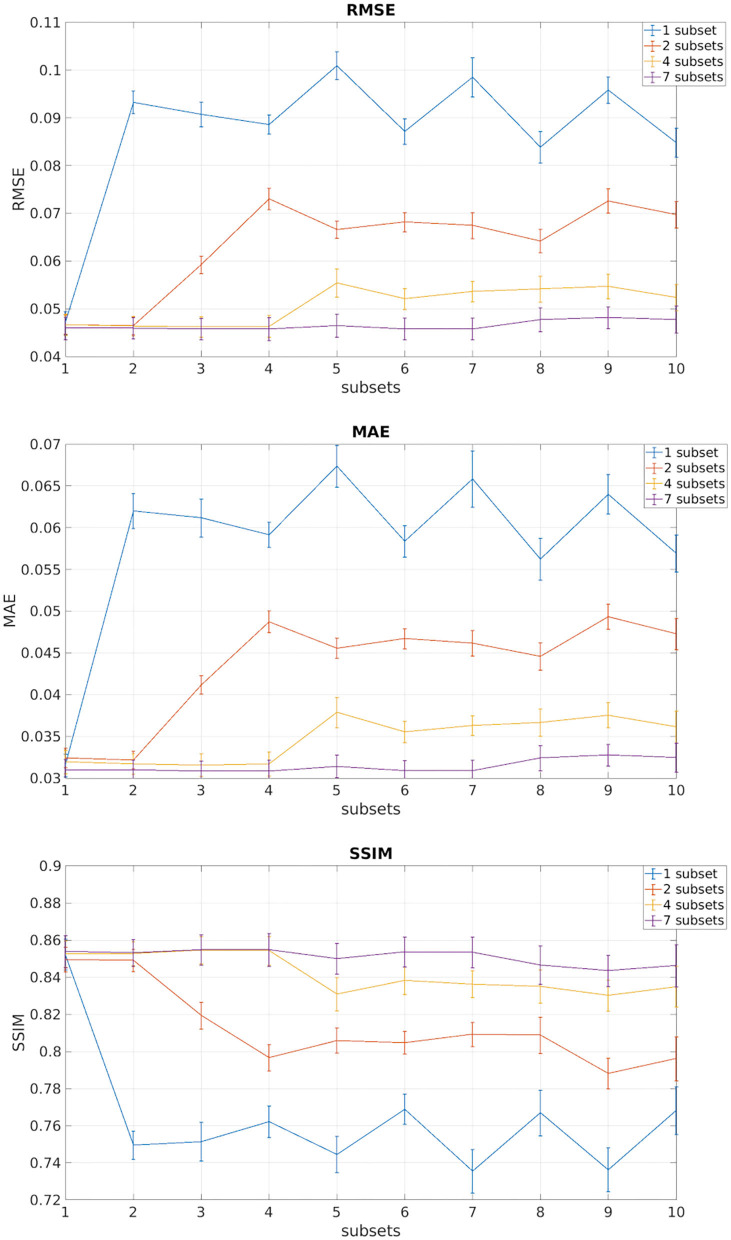
In each of the plots are reported the mean and standard deviation of the three metrics root mean square error (RMSE), mean absolute error (MAE), and structural similarity index measure (SSIM) of the test subjects for each of the 10 DW subsets. Each different color indicates the number of DW subsets (1, 2, 4, 7) used for training the network.

Figure 3 shows the plots of the three metrics: RMSE, MAE, and SSIM. In each figure, the mean and the standard deviation across the 11 test subjects, for each of the 10 DW subset, are shown for each of the four networks obtained with an increasing number of training subsets (1, 2, 4, and 7).

We observed that, for all performance metrics, the gap between metrics calculated when using the same DW volumes as the training subset/s and when using any of the DW subsets decreases with the increase in the number of DW subsets used for training. We selected the best performing network as that trained with seven DW subsets. The code for the network is publicly available at https://github.com/marta-gaviraghi/diffusion_FA.

Table 1 shows the three metrics: the RMSE, the MAE, and the SSIM on the test set for the best performing network when considering all DW subsets (all), only the DW subsets with DW directions equivalent to those used for training (training), and only the subtests that were different from the DW directions used for training (test).

**Table 1:** Performance metrics in HCP dataset.

	**10 SUBSETS (all)**	**7 SUBSESTS (training)**	**3 SUBSETS (test)**
RMSE	0.047 ± 0.002	0.046 ± 0.002	0.048 ± 0.003
MAE	0.031 ± 0.001	0.031 ± 0.001	0.033 ± 0.002
SSIM	0.851 ± 0.008	0.853 ± 0.007	0.845 ± 0.009

*For each metric, root mean square error (RMSE), mean absolute error (MAE), and structural similarity index measure (SSIM), mean and standard deviations of all test subjects are reported for (1) FA estimated using all the DW subsets, in turns, as input (all); (2) the DW subsets with b-vectors equivalent to those used for training (training); and (3) the DW subsets with b-vectors different from those used for training (test)*.

Table 2 shows the performance metrics calculated for the 11 HCP test subjects between FA GT and both the FA obtained with the standard model-fitting method from a subset of 10 DW volumes (Reduced STANDARD) and FA obtained with the network (NETWORK).

**Table 2:** Table reporting each performance metric's mean and standard deviation on fractional anisotropy (FA) values of test subjects.

	**RMSE**	**MAE**	**SSIM**
GT vs. Reduced STANDARD (Whole Brain)	0.325 ± 0.012	0.247 ± 0.012	0.242 ± 0.014
GT vs. Reduced STANDARD (White Matter)	0.245 ± 0.015	0.189 ± 0.013	0.379 ± 0.018
GT vs. NETWORK (Whole Brain)	0.046 ± 0.002	0.031 ± 0.001	0.854 ± 0.009
GT vs. NETWORK (White Matter)	0.046 ± 0.002	0.035 ± 0.002	0.904 ± 0.084

*The first two rows show metrics between ground truth (GT) FA and FA from 10 diffusion-weighted (DW) volumes using the standard model-fitting method (Reduced STANDARD); the first row refers to whole brain while the second row refers to white matter (WM) voxels only. The last two rows show performance metrics between GT and FA from 10 DW volumes using our network (NETWORK); the third row refers to the whole brain while the fourth to WM voxels only*.

The RMSE between GT and NETWORK FA and the RMSE obtained between GT and Reduced STANDARD FA were significantly different (p = 8 x 10^−5^).

### Clinical adoption potential

**Figure 4:**
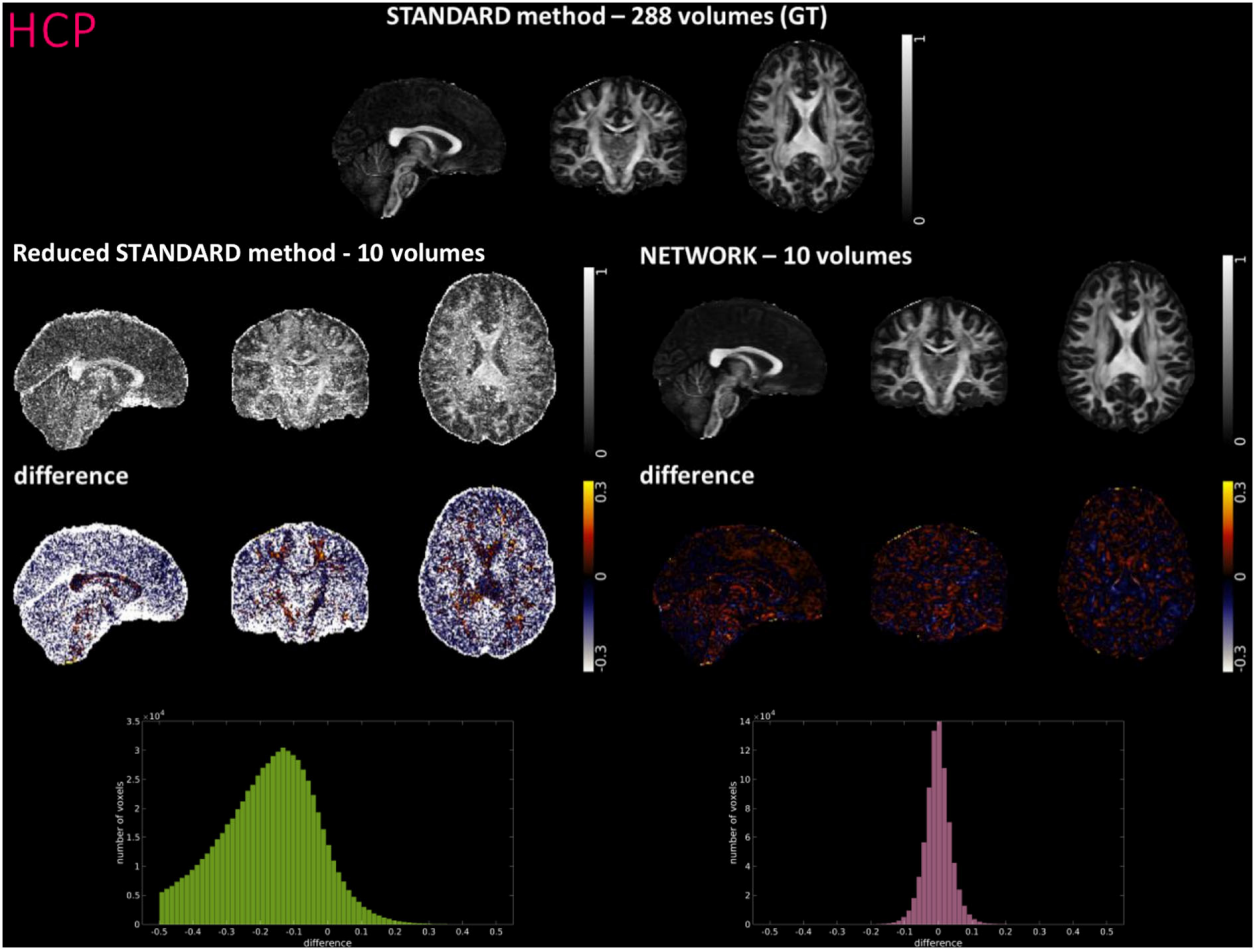
For a random subject from the Human Connectome Project (HCP), each fractional anisotropy (FA) map is shown: the standard FA map calculated with model fitting from all diffusion weighted (DW) volumes [STANDARD method−288 volumes (GT)], the standard FA map calculated with model fitting from a DW subset of 10 volumes (Reduced STANDARD method-10 volumes), and the network FA map from a DW subset (NETWORK-10 volumes). The “difference” shows the voxel-wise difference between GT FA and either the (Reduced STANDARD methods−10 volumes) FA or the (NETWORK−10 volumes) FA maps. At the bottom, there is the histogram of the differences. All color bars have arbitrary units (a. u.).

**Figure 5:**
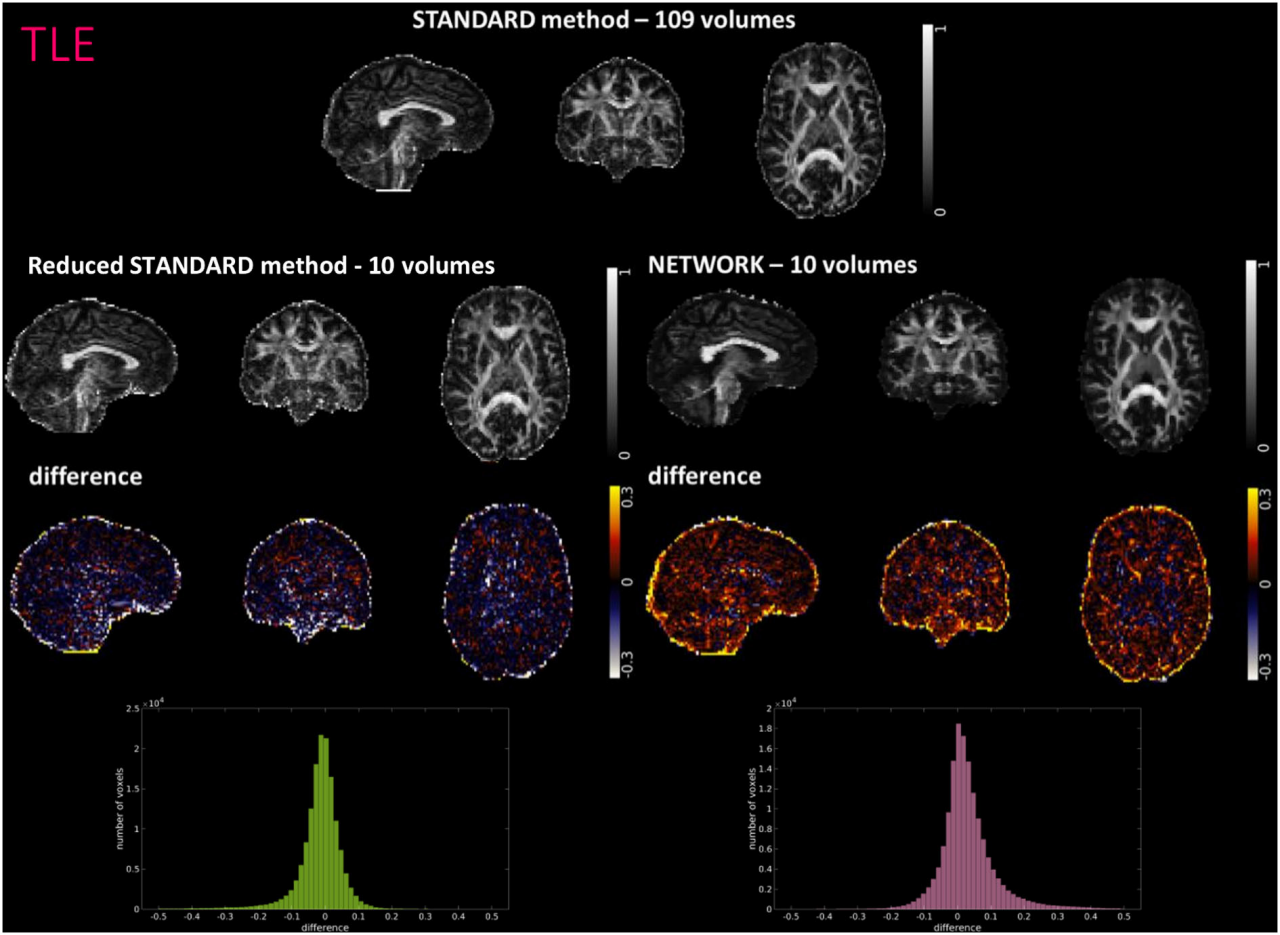
For a random subject from the temporal lobe epilepsy (TLE) study, each fractional anisotropy (FA) map is shown: the standard FA map calculated with model fitting from all diffusion-weighted (DW) volumes (STANDARD method−109 volumes), the standard FA map calculated with model fitting from a DW subset of 10 volumes (Reduced STANDARD method-10 volumes), and the network FA map from a DW subset of 10 volumes used as input (NETWORK-10 volumes). The “difference” shows the voxel-wise difference between the (STANDARD method−109 volumes) FA and either the (Reduced STANDARD methods−10 volumes) FA or the (NETWORK−10 volumes) FA maps. At the bottom, there is the histogram of the differences. All color bars have arbitrary units (a. u.).

**Figure 6:**
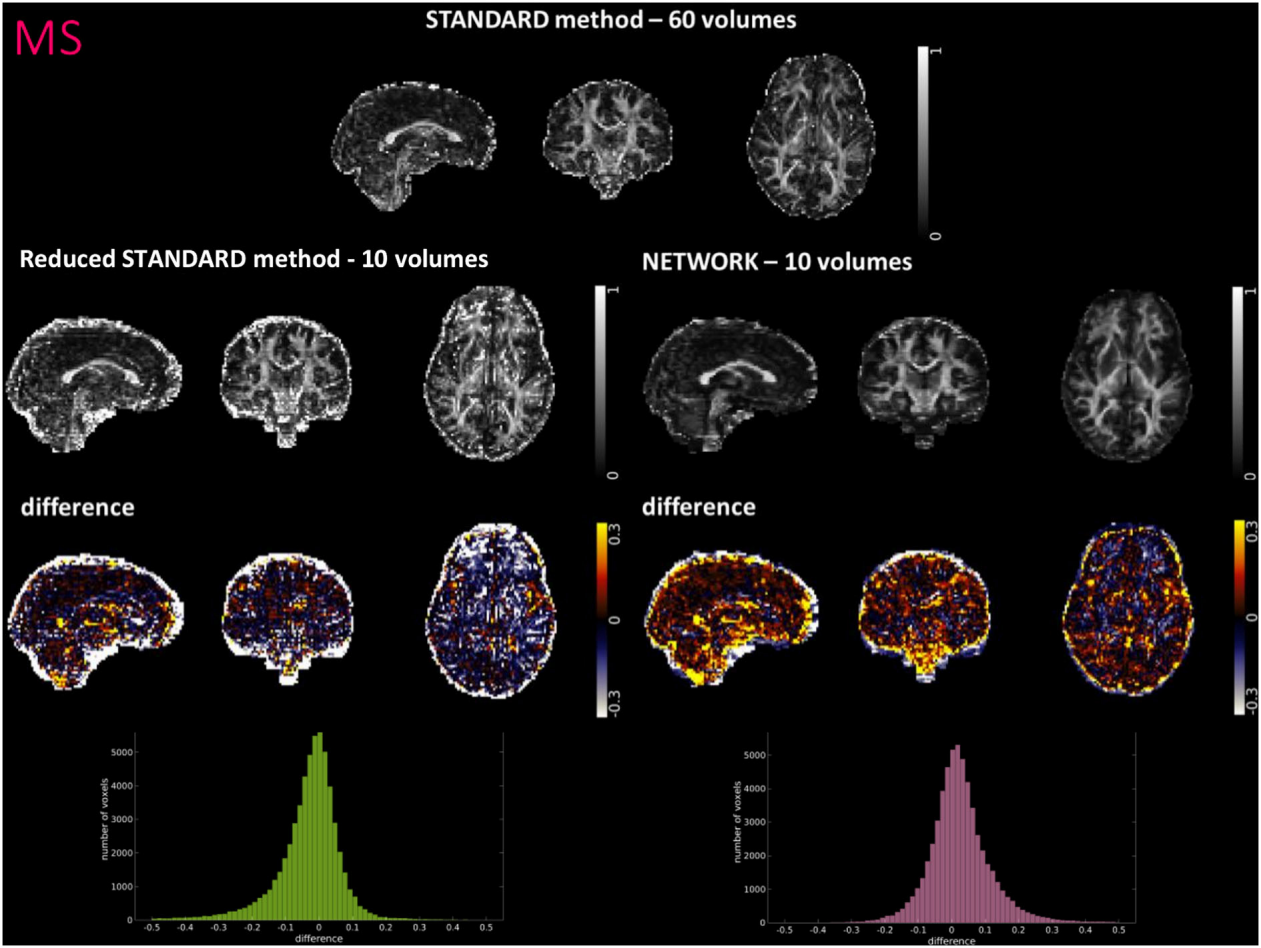
For a random subject from multiple sclerosis (MS) study, each fractional anisotropy (FA) map is shown: the standard FA calculated with model fitting from all diffusion weighted (DW) volumes (STANDARD method−60 volumes), the standard FA calculated with model fitting from a DW subset of 10 volumes (Reduced STANDARD method−10 volumes), and the network FA from a DW subset of 10 volumes used as input (NETWORK−10 volumes). The “difference” shows the voxel-wise difference between the (STANDARD method−60 volumes) FA and either the (Reduced STANDARD methods−10 volumes) FA or the (NETWORK−10 volumes) FA maps. At the bottom, there is the histogram of the differences. All color bars have arbitrary units (a. u.).

Figures 4–6 show FA maps for a random subject from the HCP, TLE, and MS datasets, respectively. In each figure, the first row shows the FA calculated with all the DW volumes using the standard model-fitting method. The second row shows the FA calculated from only 10 DW volumes: on the left, there is FA obtained with the standard model-fitting method, and on the right, the FA is obtained with the best performing network. The third row shows the difference image between the first two rows. The fourth row shows the histogram of the difference between the first two rows (GT FA minus Reduced STANDARD FA from only 10 DW volumes).

**Figure 7:**
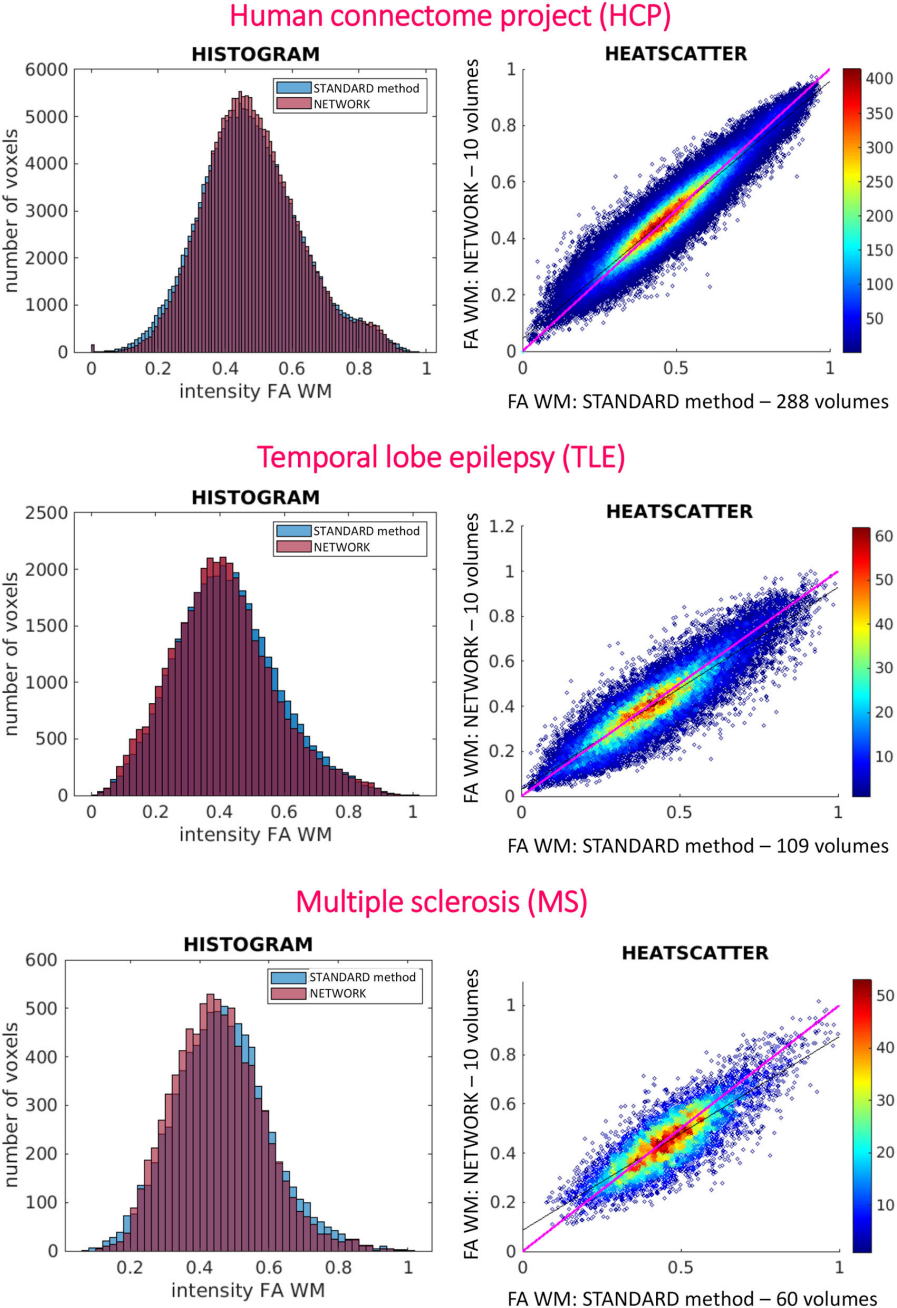
On the left are reported the histograms of fractional anisotropy (FA) distributions in white matter (WM) for the three used datasets [Human Connectome Project (HCP), temporal lobe epilepsy (TLE), and multiple sclerosis (MS)]. On the right are reported the heatscatter plots showing pairwise voxel correspondence between FA maps calculated in different ways (STANDARD method and NETWORK); the bisector of the scatterplots, for reference, is shown in pink.

Histograms and heatscatter plots are shown in Figure 7. On the left-hand side for each dataset (HCP, TLE, and MS), we showed for a single random subject the overlap of the WM FA histogram obtained with the STANDARD method with the histogram of the NETWORK WM FA. On the right-hand side, heatscatter plots are reported for each dataset to see how similar pairs of WM voxels values are when extracted from the two FA maps i.e., how close the points are to the bisector.

**Figure 8:**
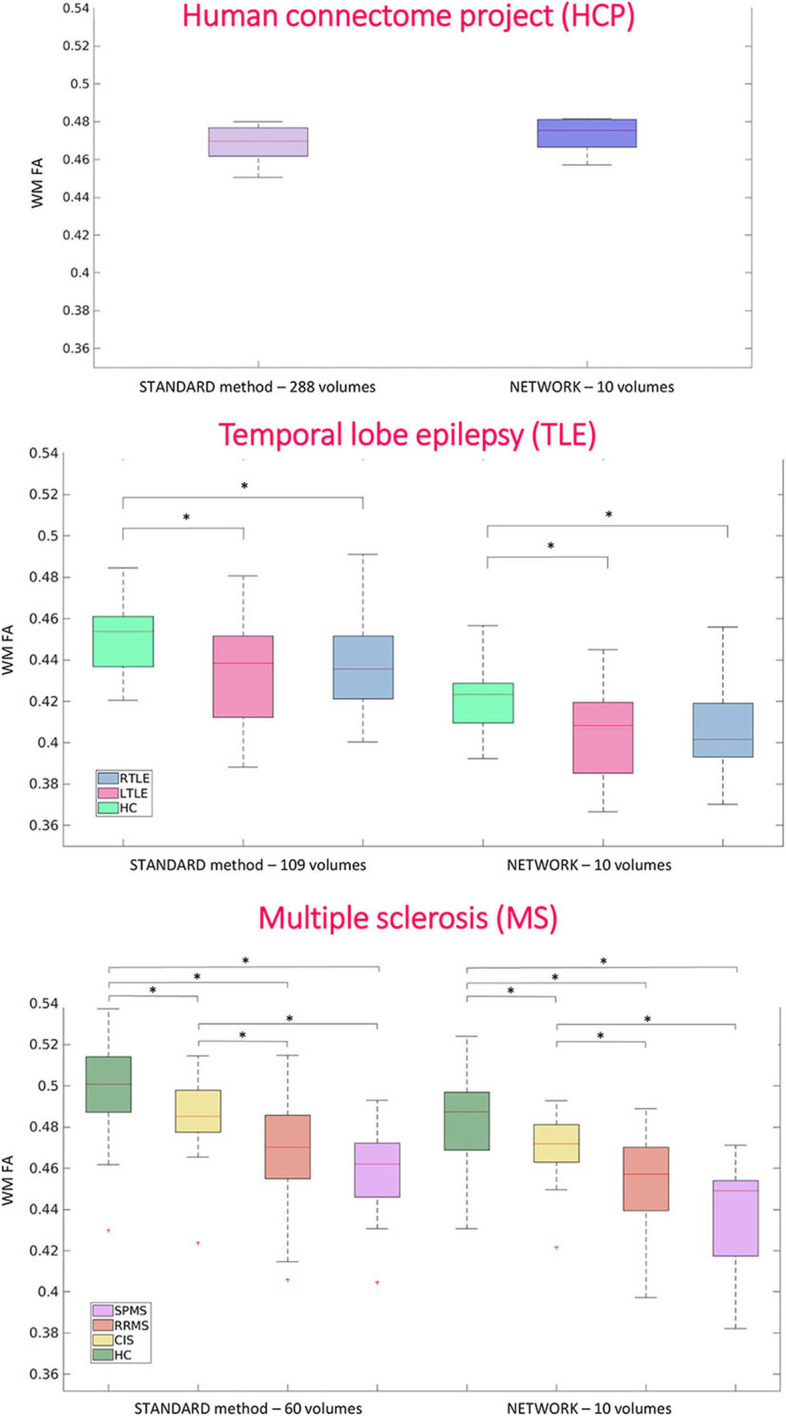
Boxplots of white matter (WM) fractional anisotropy (FA) mean values for each dataset. In the first plot, the boxplots refer to the Human Connectome Project (HCP) test subjects. Plots show the average of WM FA calculated with the standard model-fitting method on the entire DW dataset as ground truth (STANDARD method−288 volumes) and of WM FA values obtained with the network and a subset of 10 DW volumes as input (NETWORK−10 volumes). In the second plot, the boxplots refer to the temporal lobe epilepsy (TLE) subjects divided by groups: Healthy Control (HC), Left TLE (LTLE), and Right TLE (RTLE), considering the mean value of white matter fractional anisotropy of the ground truth (STANDARD method−109 volumes) and of the network (NETWORK−10 volumes). Significant differences are indicated with an asterisk (*p* < 0.05). The boxplots of the WM FA (or NAWM) refer to multiple sclerosis (MS) subjects divided by group healthy control (HC), clinical isolated syndrome (CIS), relapsing—remitting MS (RRMS), and secondary progressive MS (SPMS) considering the mean value white matter fractional anisotropy of the ground truth (STANDARD method−60 volumes) and of the network (NETWORK−10 volumes). Significant differences are indicated with an asterisk.

For performing group-level analysis and assessing clinical sensitivity, Figure 8 shows boxplots of WM FA values for each dataset. For the HCP dataset, they are calculated on the 11 test subjects either with GT (STANDARD method-−288 volumes) or with the network with a subset of 10 DW volumes used as input (NETWORK−10 volumes). For the TLE dataset, we found the same difference (*p* < 0.05) between HC and LTLE and between HC and RTLE when using either standard WM FA (STANDARD−109 volumes) or the network (NETWORK−10 volumes). For the MS dataset, we found the same differences (*p* < 0.05) in NAWM FA when comparing HC and CIS, HC and RRMS, HC and SPMS, CIS and RRMS, and CIS and SPMS using either standard FA (STANDARD−60 volumes) or the network (NETWORK−10 volumes).

**Figure 9:**
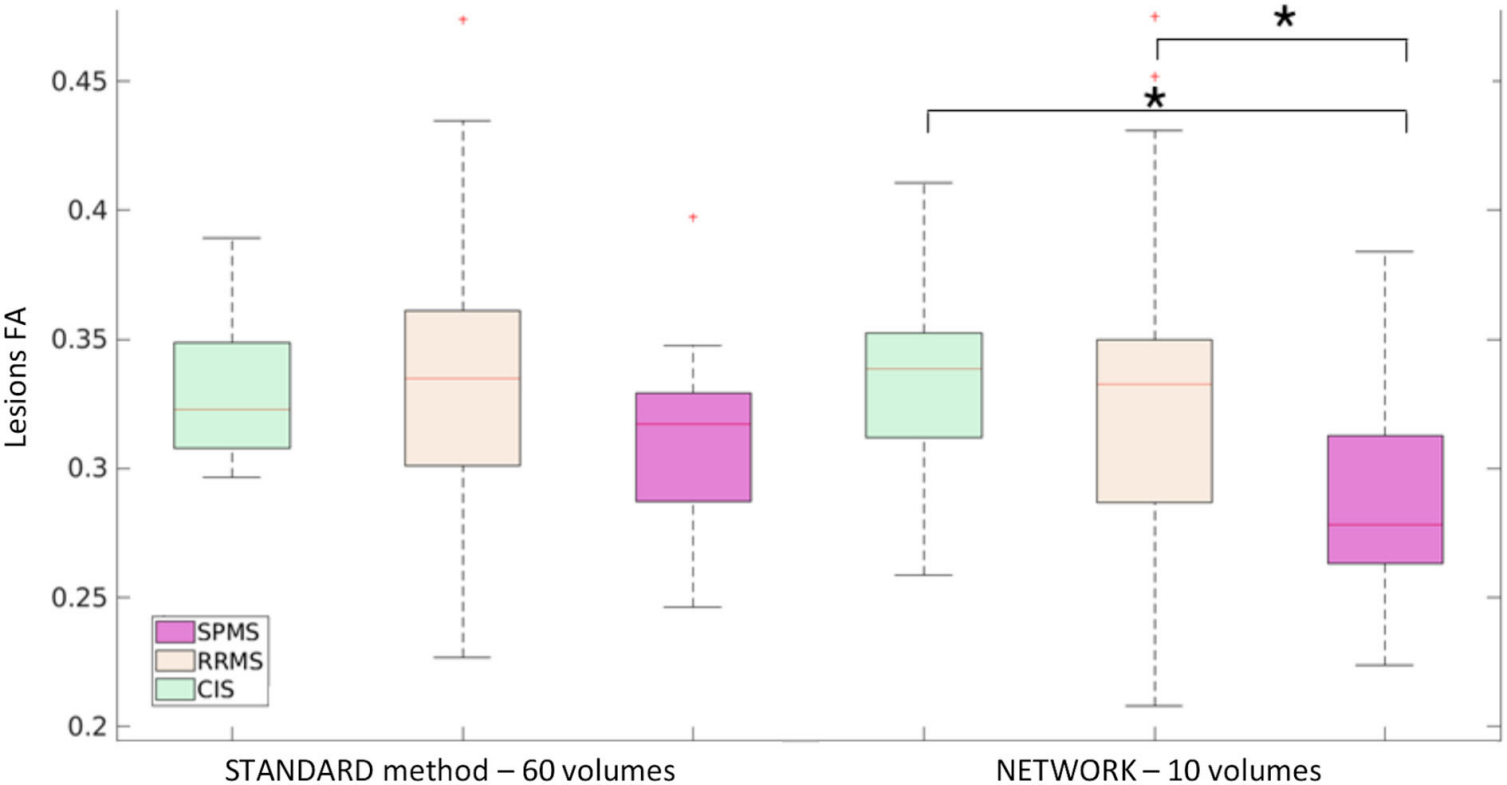
Boxplot of mean fractional anisotropy (FA) values in lesions for MS patients: subjects divided by group clinical isolated syndrome (CIS), relapsing—remitting MS (RRMS), and secondary progressive MS (SPMS) considering the mean value lesion FA of the ground truth (STANDARD method−60 volumes) and of the network (NETWORK−10 volumes). Significant differences are indicated with an asterisk (*p* < 0.05).

For the MS dataset, a boxplot of FA in lesions is reported in Figure 9. We found significant differences (*p* < 0.05) between mean lesion values when comparing CIS and SPMS or RRMS and SPMS with network FA. No significant differences were found in the lesion FA calculated using the standard method of model fitting using all volumes.

**Figure 10:**
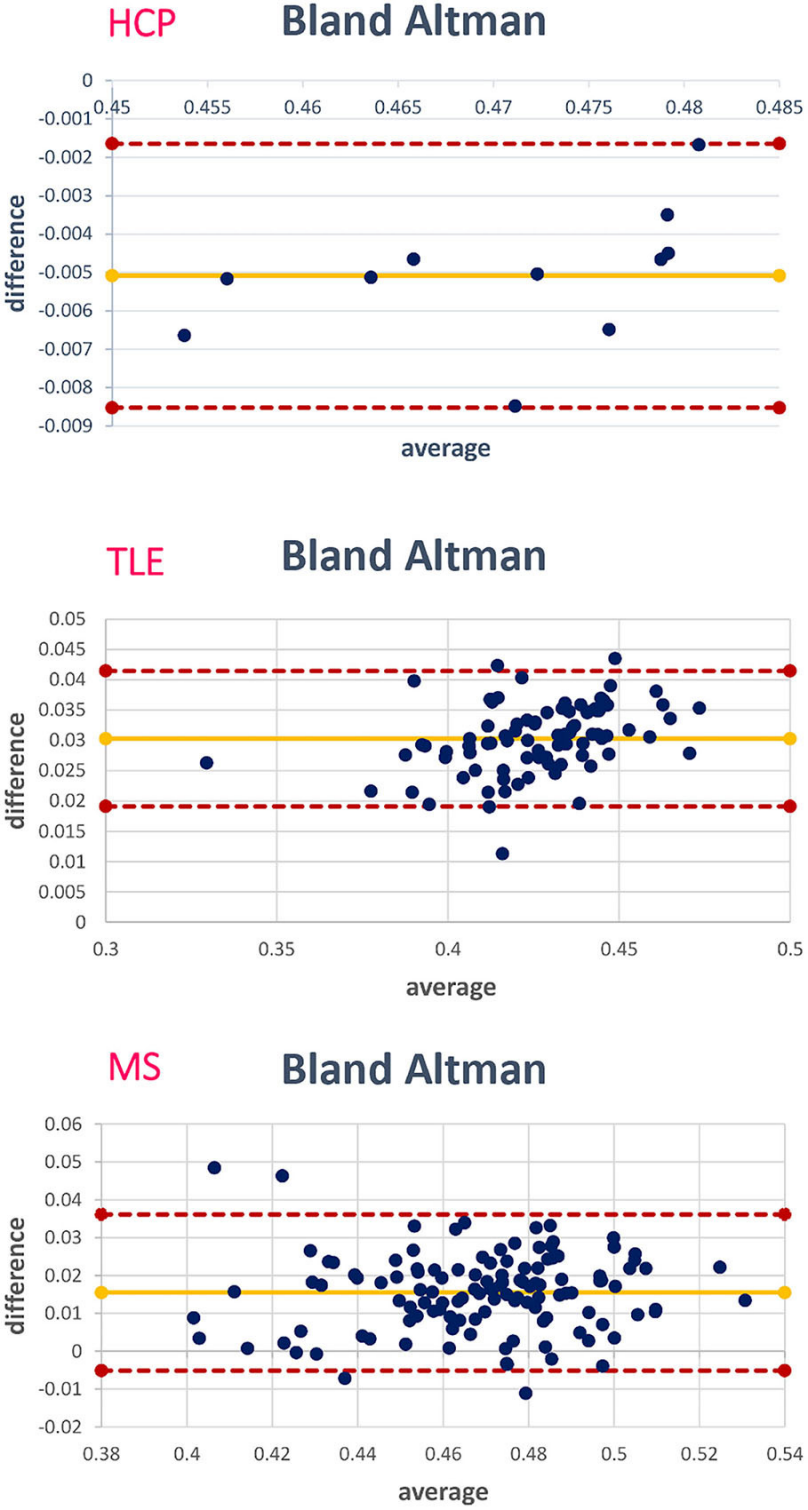
For each data set, the corresponding Bland–Altman plot is shown; each blue dot corresponds to a subject. The yellow line indicates the mean of the difference between the white matter (WM) fractional anisotropy (FA) STANDARD method and NETWORK WM FA. The red lines indicate the limits of agreement (average difference ± 1.96 standard deviation of the difference).

Figure 10 shows the Bland–Altman plots of the mean values of WM FA between the STANDARD method and NETWORK for each dataset.

## Discussion

We implemented a DL network capable of obtaining FA from a reduced set of 10 DW volumes, which could be acquired in 1 min, while retaining or enhancing image quality thanks to the fact that a DL network trained on high-quality data can contribute to image quality transfer when applied to a lower input (Alexander et al., 2017). We also generalized our network to be independent of the DW scheme acquired. We demonstrated generalizability by applying it to existing clinical datasets, acquired on different scanners from different vendors, with different geometry parameters, different DW directions, and one dataset with a different b-value than that of the training dataset.

During training, we only input diffusion images with b-values equal to 1000 s/mm^2^ because this is the value suggested for optimal white matter DT sensitivity (Basser and Jones, 2002; Jensen and Helpern, 2010). We also wanted our method to be applied in clinical settings to reduce the acquisition time for calculating the FA and fitting the diffusion tensor, where the most common b-value is 1000 s/mm^2^.

Our network was trained on a high-resolution dataset and applied to two completely different datasets from two clinical research studies in TLE and MS. In order to create a generalized network that is as independent as possible from the diffusion-encoding directions of the data used for training, we gave the network several different subsets of DW volumes as input. For model fitting, the choice of diffusion gradient directions is important: the optimal selection is the one that provides the most uniform sampling of the DW 3D spherical space (Mori and Tournier, 2014). If we were to train the network only on a single subset of the same nine DW directions, there would be a risk that the network would depend on these specific directions.

For the HCP test subjects, the NETWORK FA (RMSE = 0.046) was much closer to the GT than the Reduced STANDARD method FA (RMSE = 0.325), indicating that the network provides a clear advantage over the simple DT model fitting of data acquisition with a reduced DW protocol. Qualitatively, the NETWORK FA maps calculated using independent clinical datasets show less noisy images than the FA calculated using the STANDARD method FA (Figures 5, 6) and it provides a clear benefit from a simple model fitting of the reduced 10 DW volume dataset. It is to note, however, that, if only 10 diffusion volumes are used, a constrained fitting with non-linear least squares, such as log-Cholesky, could potentially lead to better results compared to weighted least squares, at the cost of a longer processing time (Koay, 2011).

The histograms of the difference between the FA calculated with the STANDARD method and NETWORK FA, for all three datasets, have a symmetrical distribution, meaning that the network FA shows that the negative and positive values are random (Figures 4–6). There is a clear improvement when considering the distribution of the HCP data with respect to Reduced STANDARD method FA from just the 10 volumes.

At the individual subject level, the distribution of FA in WM voxels was maintained when using the network FA for all datasets, whether they were acquired with the identical DW and geometrical protocol as the training data or on different scanners with different spatial resolution and DW schemes (Figure 7). Indeed, the histograms of WM voxels from the FA obtained with the network follow the same distribution as the WM GT FA. This is also confirmed when looking at the data with pairwise voxels as the heatscatter plots show that the WM FA values are distributed close to the bisector (Figure 7). It is worth noting that, when we extracted the subset of nine DW b-vectors from the TLE and MS data, we did not try to match the directions of one of the HCP DW subsets, but we simply extracted it from the TLE and MS DW scheme files the most uniformly distributed DW scheme of 9 b-vectors, using Camino (Cook et al., 2005). These results are very promising as they show a high generalizability of our chosen trained network.

Most importantly, the network FA retained, and possibly enhanced, the properties of the standard FA calculated with all DW volumes, including sensitivity to pathology.

At the group level, the significant pathological differences between TLE and MS sub-groups found when comparing standard FA values remained significant when using the FA estimated by the network.

In both datasets, there is a reduction of WM FA (or NAWM FA) in patients compared to controls, which is in line with previous literature on TLE and MS diseases (Horsfield, 2001; Saksena et al., 2008; Gross, 2011). Moreover, the network FA can find statistically significant differences between lesion FA in different MS sub-groups: lesions of patients with SPMS have a reduced FA compared to lesions of CIS and lesions of RRMS. These group differences are also present in the FA calculated in the standard way with all DW volumes, although here they do not reach statistical significance. This could be because the network introduces a sort of bias in the lesions as the network was trained only on healthy tissue or it could be because the network creates a higher resolution FA map that is more sensitive to pathology. This could be validated in future studies assessing the clinical relevance of the network lesion FA in terms of its correlation with neurological scores.

However, the significance found with the network is in line with the clinical data: SPMS represents the most advanced stage of MS, and therefore, their lesions are also more disrupted in terms of their microstructure than in other stages of the disease (Preziosa et al., 2011). The FA calculated with the network seems to be able to find these differences.

One limitation is that, in order to give DW volumes as input to the network, they must always have the same geometrical properties (input and output matrix size), and therefore, it is necessary to resample the images of the dataset of interest to the resolution with which the network was trained (HCP). Future studies should consider incorporating a pre-processing DL network that could learn to perform this operation.

Here, we have adapted a 2D U-net architecture because diffusion weighted images of brain slices already include all possible combinations of tissue types and microstructure architecture that define the FA contrast. Besides, 2D data are much easier to handle and 2D U-net is faster to train; nevertheless, future studies could be extended to a 3D architecture that also takes into account neighboring voxels in the third spatial dimension. We can also try to explore different architectures such as pix2pix (Isola et al., 2017) or CycleGAN (Zhu et al., 2017). Notably, recent work by Li et al. (2020) found that CT to MRI/MRI to CT image synthesis using U-net produced images with more favorable MAE, SSIM, and PSNR compared with CycleGAN.

Acquiring only 10 DW volumes greatly reduces acquisition time. In our case, considering the three datasets, the acquisition of 10 volumes took less than a minute for an HCP subject, about 1:50 min for a TLE subject, and about 2:30 min for a MS subject. In our clinical datasets, the TR was longer than for the HCP protocol because multiband excitation was not available. Using a multiband protocol, TR could be set to TR <6s, and the acquisition time could be shorter than 1 min; therefore, reconstructing FA with our network could enable the adoption of FA in a clinical setting, while retaining sensitivity to pathology.

For example, in traumatic head injuries, FA has been reported to change rapidly in the acute, subacute, and chronic phases after the injury and may correlate with cognitive impairment (Veeramuthu et al., 2015); currently, FA is not used clinically because of the longer acquisition time compared to DW scans, but our network could provide an appealing method for assessing FA clinical validity.

When no DW acquisitions are possible, Gu et al. (2019) approach to calculate FA from the T1-w scans may be the only available option, but it is possible to acquire 1 min of extra images, and we believe that acquiring DW data for our network could provide better pathological sensitivity.

Future works will aim to train the network to reconstruct the full DT, or other diffusion-derived maps, for a complete assessment of tissue microstructure and anisotropy.

## Conclusion

The proposed network can extract FA from a reduced set of 10 DW volumes, not only on test data with identical acquisition properties as the training data but also on test data with different diffusion-encoding directions and, most importantly, on data acquired on different scanners, with different DW directions and different b-values. The network FA retained the properties of the standard FA calculated with model fitting using all available DW volumes and retained, possibly enhancing, sensitivity to pathology. With our network, a 1-min FA protocol could be adopted as the standard for brain MRI protocols in clinical settings, generating data that could then be assessed radiologically for clinical indications.

## Data availability statement

Publicly available datasets were analyzed in this study. This data can be found here: https://www.humanconnectome.org/study/hcp-young-adult.

## Ethics statement

The studies involving human participants were reviewed and approved by NRES Committee London - City Road and Hampstead and the Local Ethic Committee of the IRCCS Mondino Foundation. The patients/participants provided their written informed consent to participate in this study.

## Author contributions

CGW-K, BK, AR, and MG contributed to conception and design of the study. PV, WB, and CGW-K acquired the MRI clinical data. MG performed the analysis and CGW-K, BK, AR, FPa, and FPr provided support and guidance with data analysis and interpretation. MG and CGW-K wrote the manuscript. All authors contributed to manuscript revision, read, and approved the submitted version.

## Funding

Data were provided [in part] by the Human Connectome Project, WU-Minn Consortium (Principal Investigators: David Van Essen and Kamil Ugurbil; 1U54MH091657) funded by the 16 NIH Institutes and Centers that support the NIH Blueprint for Neuroscience Research; and by the McDonnell Center for Systems Neuroscience at Washington University. 3TLE is a multicentric research project granted by Italian Health Ministry (NET2013-02355313): Magnetic resonance imaging in drug-refractory temporal lobe epilepsy: standardization of advanced structural and functional protocols at 3T, to identify hippocampal and extra-hippocampal abnormalities. CGW-K receives funding from the UK MS Society (#77), Wings for Life (#169111), BRC (#BRC704/CAP/CGW), MRC (#MR/S026088/1), Ataxia UK. CGW-K is a shareholder in Queen Square Analytics Ltd. BK and FPr are supported by the NIHR Biomedical Research Centre at UCL and UCLH.

## Conflict of interest

The authors declare that the research was conducted in the absence of any commercial or financial relationships that could be construed as a potential conflict of interest.

## Publisher's note

All claims expressed in this article are solely those of the authors and do not necessarily represent those of their affiliated organizations, or those of the publisher, the editors and the reviewers. Any product that may be evaluated in this article, or claim that may be made by its manufacturer, is not guaranteed or endorsed by the publisher.
